# A Retrospective Analysis of Demographic, Clinical, and Postoperative Data of All Cervical Spine Surgeries Performed at a Spine Center: Our Experience of 18 Years

**DOI:** 10.7759/cureus.99136

**Published:** 2025-12-13

**Authors:** Bharat R Dave, Sandesh Subhash Agarawal, Mahesh Sagar, Mirant B Dave, Shivanand C Mayi, Ravi Ranjan Rai, Ajay Krishnan, Mikeson Panthackel, Amritesh Singh

**Affiliations:** 1 Spine Surgery, Stavya Spine Hospital and Research Institute, Ahmedabad, IND; 2 Spine Surgery, Sri Devaraj Urs Academy of Higher Education and Research, Kolar, IND; 3 Spine Surgery, Shree Narayana Hospital, Raipur, IND

**Keywords:** cervical disc prolapse, cervical spine disorders, degenerative cervical myelopathy, epidemiology india, spine surgery outcomes

## Abstract

Introduction: Degenerative cervical myelopathy (DCM) and cervical disc pathology are significant causes of neurological disability worldwide, with an estimated hospital-based incidence of DCM of approximately 7.4 per 100,000 persons per year and a population prevalence of symptomatic DCM around 2-3%, while asymptomatic spinal cord compression may be present in up to 24% of adults on imaging. Indian demographic, anatomical, and socioeconomic factors uniquely influence disease patterns and treatment outcomes, yet comprehensive local data remain scarce.

Materials and methods: This retrospective cohort study analyzed 2,667 patients who underwent cervical spine surgery at a tertiary spine center in India between January 2004 and March 2025. Demographics, diagnostic distributions, perioperative complications, and functional outcomes, including Oswestry Disability Index (ODI) and Numeric Rating Scale (NRS) scores for neck pain, were evaluated. Surgical approaches varied per pathology and surgeon preference. Statistical analyses assessed pre- and postoperative differences with paired t-tests.

Results: The cohort predominantly comprised males (2,006/2,667; 75.2%) and patients aged 45-65 years (1,266/2,667; 47.5%). Cervical spondylotic myelopathy (CSM, 1,098/2,667; 41.2%) and cervical prolapsed intervertebral disc (CPID, 670/2,667; 25.1%) were the leading diagnoses. Significant postoperative improvements were observed in ODI and NRS scores across all major pathologies; for example, CSM patients showed ODI reductions from 42 ± 10 to 16 ± 3 (p < 0.001) and NRS pain score improvement from 6.2 ± 1.1 to 2.7 ± 0.7. The perioperative complication rate was low (90/2,667; 3.38%), with dural tear as the most frequent adverse event (31/2,667; 1.16%). Other complications included fever (16/2,667; 0.6%), surgical site infection (9/2,667; 0.34%), neurological worsening (4/2,667; 0.15%), and a rare mortality rate (2/2,667; 0.08%).

Conclusions: Cervical spine surgery in this Indian cohort provides significant functional and symptomatic relief with an acceptable safety profile. The demographic and diagnostic trends mirror global patterns, adapted to regional anatomical and socioeconomic contexts. These findings support tailored surgical strategies and improved spine care delivery in India.

## Introduction

Degenerative cervical myelopathy (DCM) and cervical disc pathology are significant causes of neurological disability worldwide, with an estimated hospital-based incidence of DCM of approximately 7.4 per 100,000 persons per year and a population prevalence of symptomatic DCM around 2-3%, while asymptomatic spinal cord compression may be present in up to 24% of adults on imaging [1,2]. Among the various cervical pathologies, degenerative cervical myelopathy (DCM) is widely recognized as the most common cause of spinal cord dysfunction in adults. Its most frequent form, cervical spondylotic myelopathy (CSM), develops gradually due to age-related disc degeneration, osteophyte formation, loss of disc height, and thickening of supporting ligaments that collectively narrow the cervical spinal canal [3,4]. As life expectancy increases and populations age, the incidence of degenerative cervical diseases continues to rise, resulting in greater healthcare utilization, increased surgical demand, and broader socioeconomic impact [5].

Traumatic cervical spine injuries also represent a major contributor to morbidity. In many developing regions, including India, road traffic accidents and falls remain the leading causes. These injuries often present with varied patterns of instability, neurological compromise, and potential for long-term disability if not treated promptly [6]. The burden of cervical spine disease in India is shaped by several region-specific factors, including population density, occupational strain, cultural habits, and variations in access to healthcare services [7,8]. In addition, anatomical and morphometric studies have shown that Indian patients often exhibit smaller pedicle dimensions, narrower laminae, and other structural features that differ from Western populations [9]. These differences make it necessary to adapt surgical planning, choose appropriate implant sizes, and consider modified operative techniques to improve safety and outcomes [10].

Management of cervical spine disorders in India is further influenced by unequal distribution of specialized spine centers, variable socioeconomic status, and challenges related to timely diagnosis in resource-limited settings. Patients often present late in the disease course, which may increase the severity of symptoms and the complexity of surgical intervention. These systemic factors continue to affect outcomes and highlight the need for data-driven improvements in clinical practice [11,12].

Although some institutional and registry-based data exist in the Indian context, comprehensive studies covering the full spectrum of cervical spine disorders are limited. Most available reports focus on specific pathologies or small sample sizes, creating a gap in understanding large-scale patterns, complication rates, and outcome trends across diverse patient groups [13].

This retrospective cohort of 2,667 cervical spine patients helps bridge these knowledge gaps by offering comprehensive data on demographic profiles, diagnostic patterns, perioperative events, and surgical outcomes. By evaluating a large and heterogeneous patient population, the study provides meaningful insights that can inform clinical decision-making, enhance surgical planning, and facilitate the development of context-specific strategies for cervical spine care in India.

## Materials and methods

Study design

This retrospective cohort study was conducted at Stavya Spine Hospital and Research Centre, a tertiary care spine institute in India. All consecutive cervical spine surgeries performed from 2004 to 2025 were analyzed. This research was approved by the Institutional Ethics Committee of Stavya Spine Hospital and Research Centre (Approval No: SSHRI/CS/NS/Retro 18Cx/BRD/47/01-22). Written (or verbal, if applicable) informed consent was obtained from all participants or their legal guardians prior to surgery and follow-up, in accordance with the Declaration of Helsinki.

Study population and sample size

Eligible participants included all adults (aged >18 years) who underwent cervical spine surgery for degenerative, traumatic, neoplastic, or other pathologies within the study period. Patients were included if they had complete clinical, operative, and follow-up documentation. The most common diagnostic categories included cervical spondylotic myelopathy, cervical prolapsed intervertebral disc, foraminal spinal canal stenosis, and segmental degeneration, among others, as defined by radiological and clinical evaluation. Patients were excluded if they had incomplete records or were lost to follow-up. Of 2,704 identified cases, 2,667 (98.6%) met eligibility criteria, with 37 excluded for incomplete data or loss to follow-up.

Study measures

Demographic (age, sex, comorbidities) and clinical data were retrieved from the electronic medical record. The diagnosis was established based on clinical and radiological findings (cervical radiography, CT, and MRI) according to institutional protocol. The choice of surgical approach (anterior, posterior, or combined) was individualized. Functional outcomes were assessed with the Oswestry Disability Index (ODI) [14] and Numeric Rating Scale (NRS) [15] for neck pain, both administered preoperatively and at the most recent postoperative follow-up. Permission to administer the ODI was obtained; the NRS is public domain. Perioperative complications were prospectively recorded and included dural tears, hemorrhage, neurological deterioration, infection, postoperative fever, and mortality. Postoperative follow-up was completed in person or via telephone with a standardized questionnaire.

Patient satisfaction assessment

At the most recent postoperative follow-up, patient satisfaction was documented using a structured questionnaire administered either in-person or via telephone. The questionnaire evaluated overall satisfaction with surgical outcomes, improvement in pain and function, and willingness to recommend the procedure. Responses were recorded on a 4-point Likert scale: excellent, good, fair, or poor. This approach allowed consistent assessment of subjective outcomes across the large cohort, despite variations in follow-up timing over the 18-year study period.

Follow-up protocol

All patients were scheduled for postoperative follow-up at regular intervals, typically at six weeks, three months, six months, one year, and annually thereafter. Follow-up assessments were conducted either in-person at the spine clinic or via telephone for patients unable to attend in person. Patient-reported outcomes, clinical examinations, and complications were documented at each follow-up visit. Despite the long study period (18 years), efforts were made to contact all patients, though a small subset was lost to follow-up due to relocation, mortality, or inability to reach the patient.

Statistical analysis

Descriptive statistics were used for all baseline and outcome variables, with categorical data reported as absolute numbers and percentages (n [%]). Continuous variables are presented as mean ± standard deviation (SD) or median [IQR] as appropriate. Normality of data was assessed using the Shapiro-Wilk test and Q-Q plots. Differences in pre- and postoperative ODI and NRS scores were analyzed using paired two-tailed t-tests. The frequency of perioperative complications between subgroups was tested using the chi-square test. For all main comparisons, 95% confidence intervals and p values (<0.05 considered significant) were reported. Analyses were performed using IBM Corp. Released 2020. IBM SPSS Statistics for Windows, Version 26. Armonk, NY: IBM Corp.

## Results

A total of 2,667 patients who underwent cervical spine surgery from 2004 to 2025 were included. The cohort comprised 2,006/2,667 males (75.2%) and 661/2,667 females (24.8%). The majority of patients were aged 45-65 years (1,266/2,667; 47.5%), with full demographic distribution shown below in Table 1. 

**Table 1:** Patient Demographics Distribution of patients by age group and sex among all individuals (n=2,667) who underwent cervical spine surgery. Values are shown as numbers (n) and percentages (%).

Age Group (years)	n (%)
0–17	31 (1.2%)
18–44	649 (24.4%)
45–65	1,266 (47.5%)
>65	721 (27.0%)
Total	2,667 (100%)

Cervical spondylotic myelopathy (CSM) was the most common diagnosis (1,098/2,667; 41.2%), followed by cervical prolapsed intervertebral disc (CPID; 670/2,667; 25.1%). Other pathologies and their proportions are shown in Table 2.

**Table 2:** Diagnosis Distribution Breakdown of all primary cervical spine pathologies diagnosed among the study cohort (n=2,667). Values are n (%). CSM: cervical spondylotic myelopathy; CPID: cervical prolapsed intervertebral disc; SD: segmental degeneration; UC: unclassified/unknown; CI: cervical instability; OPLL: ossification of posterior longitudinal ligament; RS: rheumatoid spondylitis; AS: ankylosing spondylitis; ACM: Arnold Chiari malformation; SOL: space occupying lesion; CDP: cervical disc protrusion.

Diagnosis	n (%)
CSM	1,098 (41.2%)
CPID	670 (25.1%)
Fracture Cervical Spine	198 (7.4%)
Segmental Degeneration	126 (4.7%)
Unclassified/Unknown	179 (6.7%)
Instability	57 (2.1%)
CPID with Stenosis	125 (4.7%)
OPLL	17 (0.6%)
Rheumatoid Spondylitis	99 (3.7%)
Ankylosing Spondylitis	6 (0.2%)
Arnold Chiari Malformation	4 (0.1%)
SOL/SOLID	56 (2.0%)
CDP	32 (1.2%)
Total	2,667 (100%)

All major pathology groups showed statistically significant improvements in both ODI and NRS scores. The pre- and post-operative mean ± SD scores are listed in Table 3 (ODI) and Table 4 (NRS), alongside mean differences and paired t-test p-values. All ODI and NRS differences met assumptions of normality (Shapiro-Wilk p>0.05).

**Table 3:** Oswestry Disability Index (ODI) Scores (Mean ± SD, Range) With Paired t-Test Mean ± standard deviation (SD) and range for ODI before and after surgery in major diagnostic subgroups. Includes mean difference, 95% confidence interval (CI), and paired t-test p-values. CSM: cervical spondylotic myelopathy; CPID: cervical prolapsed intervertebral disc; ACM: Arnold Chiari malformation; AS: ankylosing spondylitis; SD: segmental degeneration; SOL: space occupying lesion.

Pathology	n	Preop NRS	Postop NRS	Mean Diff.	95% CI	p-value
CSM	1,098	6.2 ± 1.1	2.7 ± 0.7	3.5	[3.4–3.6]	<0.001
CPID	670	6.8 ± 1.3	1.9 ± 0.7	4.9	[4.8–5.0]	<0.001
FSC	198	6.5 ± 1.0	2.6 ± 0.8	3.9	[3.7–4.1]	<0.001
Instability	57	6.6 ± 1.2	3.1 ± 0.9	3.5	[3.2–3.8]	<0.001
ACM	4	7.0 ± 1.0	3.6 ± 0.9	3.4	[1.8–5.0]	<0.05
AS	6	6.4 ± 1.1	2.8 ± 0.9	3.6	[2.6–4.6]	<0.05
Segmental Degeneration	126	6.7 ± 1.2	3.0 ± 0.8	3.7	[3.5–3.9]	<0.001
SOL	56	6.8 ± 1.2	3.4 ± 0.8	3.4	[3.1–3.7]	<0.05

**Table 4:** Numeric Rating Scale (NRS) for Neck Pain (Mean ± SD) With Paired t-Test Mean ± SD of NRS neck pain scores before and after surgery by diagnosis, with mean difference, 95% CI, and paired t-test p-values. CSM: cervical spondylotic myelopathy; CPID: cervical prolapsed intervertebral disc; ACM: Arnold Chiari malformation; AS: ankylosing spondylitis; SD: segmental degeneration; SOL: space occupying lesion, FSC, foraminal spinal canal stenosis.

Pathology	n	Preop NRS	Postop NRS	Mean Diff.	95% CI	p-value
CSM	1,098	6.2 ± 1.1	2.7 ± 0.7	3.5	[3.4–3.6]	<0.001
CPID	670	6.8 ± 1.3	1.9 ± 0.7	4.9	[4.8–5.0]	<0.001
FSC	198	6.5 ± 1.0	2.6 ± 0.8	3.9	[3.7–4.1]	<0.001
Instability	57	6.6 ± 1.2	3.1 ± 0.9	3.5	[3.2–3.8]	<0.001
ACM	4	7.0 ± 1.0	3.6 ± 0.9	3.4	[1.8–5.0]	<0.05
AS	6	6.4 ± 1.1	2.8 ± 0.9	3.6	[2.6–4.6]	<0.05
Segmental Degeneration	126	6.7 ± 1.2	3.0 ± 0.8	3.7	[3.5–3.9]	<0.001
SOL	56	6.8 ± 1.2	3.4 ± 0.8	3.4	[3.1–3.7]	<0.05

Ninety of 2,667 patients (3.38%) experienced perioperative complications. Dural tear was most common, affecting 31 patients (1.16%). Frequencies and percentages of all complications are outlined in Table 5.

**Table 5:** Perioperative Complications CSF: Cerebrospinal fluid

Complication	n (%)
Dural Tear / CSF Leak	31 (1.16%)
Postoperative Hemorrhage / Bleeding	2 (0.075%)
Neurological Deterioration	4 (0.15%)
Surgical Site Infection (SSI)	9 (0.34%)
Postoperative Fever	16 (0.60%)
Mortality / Death	2 (0.08%)
Other (each < 0.1%)	10 (0.38%)
Any complication	90 (3.38%)

## Discussion

Demographics and epidemiology

This large retrospective review of 2667 surgically treated cervical spine cases represents one of the largest such Indian series to date. The demographic profile reflects patterns observed in similar registries worldwide while also revealing unique regional features. Half of the study population fell between 45 and 65 years, consistent with the epidemiological consensus that degenerative cervical spine diseases are primarily disorders of middle to late adulthood [1-3,5]. This period coincides with cumulative mechanical loading and biochemical degeneration of spinal structures, eventually manifesting clinically as cervical myelopathy, radiculopathy, and pain syndromes. The fact that 27% of patients were aged above 65 emphasizes the growing geriatric spine disease burden in India, mirroring global aging trends [5]. A key finding was the marked male predominance (75.2%), consistent with prior Indian epidemiological studies [7,8]. Explanations include higher exposure to manual labor and traffic-related trauma in men, as well as sociocultural factors that delay care-seeking among women. This study revealed a pronounced male predominance (75.2%) in patients undergoing cervical spine surgery, in contrast to global series that show a more balanced sex distribution. This gender disparity likely reflects higher injury exposure among men and sociocultural barriers limiting women’s access to specialized spine care, highlighting the need for targeted public health strategies to improve awareness, access, and timely treatment for women.

Diagnostic spectrum

Patients were categorized according to their primary clinical and radiological diagnosis, which represented the main indication for surgical intervention. Secondary contributing pathologies, such as cervical disc protrusions causing cord compression in cases of cervical spondylotic myelopathy (CSM), were recorded but did not lead to multiple group assignments. This method ensured mutually exclusive diagnostic groups for prevalence and outcome analysis while acknowledging potential overlapping etiologies. Cervical spondylotic myelopathy (CSM) was the most common surgical diagnosis (41.2%), consistent with its status as the leading cause of adult spinal cord dysfunction worldwide [1,4,16,17]. Chronic cord compression in CSM arises from multilevel spondylotic degeneration, disc protrusions, osteophytes, and ligamentous hypertrophy [3,4,16]. Cervical prolapsed intervertebral disc (CPID) accounted for 25.1% of cases, in line with global registry data [12,18]. Surgical decompression in radiculopathy is associated with marked improvements in pain and functional disability [12,18]. Other less frequent diagnoses included foraminal stenosis, ossification of the posterior longitudinal ligament (OPLL), ankylosing spondylitis, acute cervical myelopathy, and space-occupying lesions such as tumors or infections [13-15]. The relatively higher prevalence of OPLL in Indian populations compared to Western cohorts is supported by morphometric studies showing smaller vertebral dimensions and increased ligamentous ossification in Indians and other Asians [9,10,13,15]. Such anatomical differences necessitate careful surgical planning and population-specific instrumentation designs [10]. Comparison with Western cohorts shows Indian patients have higher rates of ossification-related disorders and trauma sequelae, whereas North American and European populations show more chronic disc disease or rheumatoid pathology [10,13]. This emphasizes the need for population-specific treatment guidelines and implant innovations.

Functional outcomes

Surgery produced significant symptom relief and functional improvement. Postoperatively, the Oswestry Disability Index (ODI) improved by ~60%, and Numeric Rating Scale (NRS) neck pain scores declined from moderate-severe to mild levels, consistent with international analyses. Patients with CSM exhibited neurological and functional recoveries comparable to North American and Japanese registry outcomes, where anterior, posterior, and combined approaches show broadly equivalent long-term improvements [16-19,20-21]. CPID and foraminal stenosis patients experienced rapid radiculopathy relief, supporting evidence that nerve root decompression yields a durable benefit [12,18]. Even rarer conditions, ankylosing spondylitis, acute traumatic myelopathy, tumors, and infections, demonstrated meaningful postoperative improvements [14,20], validating surgical intervention in complex populations. Patient-reported satisfaction was high, with nearly 60% rating outcomes as good to excellent [22]. Delayed presentation remains a key factor in suboptimal recovery due to irreversible neural damage.

Perioperative complications and safety profile

Overall complication rate was 3.38%, favorable compared with the 3-15% range reported worldwide [23-26]. Dural tears were the most frequently encountered intraoperative complication, occurring in 31 patients (1.16%), and all cases were managed successfully [23,24]. Subgroup analysis revealed that these injuries were most common in the 45-65-year age group, which also represents the largest segment of our cohort. The majority of dural tears occurred in patients with cervical spondylotic myelopathy (CSM), likely due to multilevel spondylotic changes, adhesions, and ligamentous hypertrophy that increase the technical difficulty of decompression. These findings align with previous reports indicating that extensive degenerative pathology and multilevel surgery are risk factors for dural injury [Ref]. Postoperative fever (0.6%) and surgical site infection (0.34%) were infrequent, remaining lower than rates reported internationally [25]. Mortality was minimal (0.08%), aligning with outcomes from elective cervical surgery series [26]. Overall, these results underscore the importance of surgical expertise, standardized operative protocols, appropriately selected implants, and coordinated multidisciplinary perioperative care [24]. Recognizing patient age and underlying pathology as risk factors may further guide surgical planning, intraoperative precautions, and preoperative counseling. Delivery of high-quality cervical spine surgery with complication rates comparable to leading international centers is achievable within well-structured Indian tertiary care institutions [27].

Implications for Indian spine care

Aging demographics indicate increasing cervical surgery workload in India, highlighting the need for geriatric spine care pathways, screening campaigns, and early-intervention policies [7,28,29]. The observed gender disparity highlights underlying social inequities in access to spine care, which can be mitigated through women-focused awareness initiatives, affordable care programs, and community-based education efforts [28,29]. Anatomical differences support context-specific spinal implant design; pedicle screws, cages, and plates optimized for larger Western vertebrae may pose risks when applied to Indian morphometry [9,10,30]. Local innovations and navigation training are essential for safe stabilization. Rural access remains limited; regional spine units, telemedicine, and rural surgeon training can reduce inequities and ensure timely surgical care [31,32].

Limitations and future directions

Retrospective design introduces selection bias and variable data completeness. Limited long-term follow-up restricts assessment of late outcomes. Standardized patient-reported outcome measures were not universally applied. Future research should be prospective and multicenter, use uniform instruments (e.g., the modified Japanese Orthopaedic Association score), and incorporate extended follow-up. Population-level studies should explore genetic, nutritional, and occupational determinants of cervical pathology in Indians. Therapeutic evaluations of biologics, regenerative approaches, motion-preserving implants, and cost-effective innovations are warranted.

## Conclusions

This study highlights that cervical spine disorders in India predominantly affect middle-aged and elderly men, with degenerative conditions being the leading cause. Surgery demonstrated substantial improvements in pain and disability, with low complication rates, even in complex cases. These findings reaffirm the effectiveness and safety of surgical interventions when delivered in specialized tertiary centers and provide valuable epidemiological and clinical insights that can guide future research and aid in developing population-specific strategies for cervical spine care in the Indian context.
